# Microalbuminuria and Hypertension among Immigrants with Type 2 Diabetes: A Community-Based Cross-Sectional Study †

**DOI:** 10.3390/jpm12111777

**Published:** 2022-10-28

**Authors:** Shiryn D. Sukhram, Gustavo G. Zarini, Lemia H. Shaban, Joan A. Vaccaro, Fatma G. Huffman

**Affiliations:** 1 Department of Biology, College of Staten Island, City University of New York, Staten Island, New York, NY 10314, USA; 2 Clinical & Scientific Research, Oxford Biomedical Technologies, West Palm Beach, FL 33404, USA; 3 Department of Food Science and Nutrition, College of Life Sciences, Kuwait University, Safat, Kuwait City 13060, Kuwait; 4 Department of Dietetics and Nutrition, Robert Stempel College of Public Health and Social Work, Florida International University, Miami, FL 33199, USA

**Keywords:** cardiovascular disease, hypertension, type 2 diabetes, immigrants, microalbuminuria

## Abstract

Purpose: This study examined the association of microalbuminuria (MAU), as determined by albumin-to-creatinine ratio (ACR), with hypertension (HTN) among Turkish immigrants with type 2 diabetes (T2D) living in deprived neighborhoods of The Hague, Netherlands. Methods: A total of 110 participants, physician-diagnosed with T2D, aged ≥ 30 years were recruited from multiple sources from The Hague, Netherlands in a cross-sectional design. Systolic blood pressure (SBP) and diastolic blood pressure (DBP) were measured using automated office blood pressure equipment. Urine albumin was measured by immunoturbidimetric assay. Urine creatinine was determined using the Jaffe method. MAU was defined as ACR ≥ 3.5 mg/mmol for females and/or ACR ≥ 2.5 mg/mmol for males. Results: MAU was present in 21% of Turkish immigrants with T2D. Adjusted logistic regression analysis indicated that the odds of having MAU were 6.6 times higher in hypertensive than those that were normotensive (*p* = 0.007; 95% confidence interval [CI]: 1.19, 36.4). Conclusion: These findings suggest that HTN and MAU may be assessed as a standard of care for T2D management for this population. Prospective studies of diabetes outcomes are recommended to further verify these findings.

## 1. Introduction

Chronic kidney disease (CKD) is a worldwide problem that carries a significant risk for cardiovascular morbidity and mortality [1]. Research indicates ethnic inequalities in CKD prevalence, however data from diverse ethnic groups in Europe are limited. According to the Healthy Life in an Urban Setting (HELIUS) study, the prevalence of CKD was higher in Turkish individuals (95% CI, 6.7–9.4%) as compared with the native Dutch (95% CI, 2.3–3.7%) [2]. Additionally, when compared to the Dutch, ethnic minority males showed higher treatment rates for hypertension (HTN) and dyslipidemia whereas ethnic minority females showed similar treatment rates relative to the Dutch [3]. These findings highlight the need for further research to identify other potential causes contributing to the ethnic differences in CKD. The Turkish immigrant population has experienced stressors based on their status and race/ethnicity that have the potential to contribute toward poorer health.

A small but abnormal albumin excretion in urine is known as microalbuminuria (MAU). MAU is a widely known predictor of diabetic nephropathy, essential hypertension (HTN), and cardiovascular disease (CVD) [4,5,6]. It is important that MAU be measured in all subjects with type 2 diabetes (T2D) and HTN, so that renal and cardiovascular adverse events can be properly managed and prevented [7,8,9].

Hypertensive T2D individuals with MAU are at increased risk of developing end stage renal disease (ESRD) [10,11]. T2D is highly prevalent among ethnic minorities living in Western societies. In the Netherlands, Turkish immigrants form the largest ethnic minority group with 417,000 inhabitants according to 2020 census [12]. Research indicates that the onset of diabetes occurs one decade earlier among the Turkish when compared to the native Dutch [13,14,15,16]. Moreover, mortality from CVD in patients with T2D is higher than in the general population [7,17]. The high prevalence of T2D and CVD in the Turkish immigrant population in the Netherlands merits further assessment of diabetes complications [13,18]. The Trabzon Hypertension Study showed that HTN is common among the Turkish adult population and uncontrolled hypertensive individuals are at high risk of cardiovascular morbidity and mortality [19]. A cross-sectional observational study carried out in Turkey demonstrated that Turkish hypertensive patients were not adequately evaluated for CVD risk [20]. We therefore assessed MAU in Turkish hypertensive patients with T2D living in deprived neighborhoods of The Hague, Netherlands. The Hague, a city that is part of the Randstad metropolitan area, is vastly urbanized with a large population of Turkish immigrants (n = 8097) [21,22]. The Schilderswijk neighborhood is considered a deprived area of The Hague populated by 91.1% non-Western immigrants and 8.9% native Dutch. According to Gemeente Den Haag census, Turkish immigrants are the most prevalent inhabitants of the Schilderswijk area. Furthermore, 61.3% residents in the Schilderswijk area are aged 20–64 years, with an average age of 35 years.

Microalbuminuria is an important early indicator for diabetic nephropathy and a significant risk factor for progression to proteinuria, ESRD, and mortality. Additionally, MAU is a predictor of cardiovascular mortality, as research indicates that individuals with MAU are at four-fold increase of death from CVD, particularly among the hypertensive population [23]. Among individuals with both T2D and HTN, the presence of MAU is associated with increased prevalence of CVD [24]. Yang et al. proposed one pathway aimed at explaining the relationships among MAU, HTN, diabetes, and CVD [25]. In this pathway, hyperglycemia and HTN lead to MAU, which in turn results in hyperlipidemia, a significant risk factor for CVD. Patients with T2D are more prone to dyslipidemia. Diabetes-induced dyslipidemia may be manifested by reduced high-density lipoprotein cholesterol (HDL-c), increased low-density lipoprotein cholesterol (LDL-c), and increased triglyceride (TG) levels in the blood [26]. In patients with diabetic dyslipidemia, statin therapy has beneficial effects to cardiovascular CVD health [26]. However, other than decreasing the LDL-c levels, the management of dyslipidemia in patients with T2D continues to be challenging [27]. Diabetes, cigarette smoking, and dyslipidemia are well-known risk factors for CVD in the general population in the Netherlands [28].

The American Diabetes Association (ADA) recommends testing for MAU in individuals with T2D on initial diagnosis of the disease and every year afterwards [9]. Albumin levels, which are typically low due to retention of the protein in the bloodstream by the kidneys, can be detected using albumin-specific urine dipsticks. This method is a convenient and accurate screening tool [29]. However, some variation in urine albumin concentration can arise. To compensate for variations in urine concentration in spot-check samples, it is helpful to compare the amount of albumin in the sample against its concentration of creatinine. This is termed the albumin/creatinine ratio (ACR) and MAU is defined as ACR ≥ 3.5 mg/mmol (female) or ≥ 2.5 mg/mmol (male), or, with both substances measured by mass, as an albumin to creatinine ratio between 30 and 300 µg albumin/mg creatinine [30]. However, it is important to note that these cut-offs may not be appropriate in all situations. Men with high muscle mass and members of various ethnic groups, including Hispanics, may demonstrate higher baseline levels of albumin [31]. Additionally, research suggests that levels as low as 0.32 mg/mmol of MAU or an ACR as low as 5 µg albumin/mg creatinine may be associated with significant risk of CVD [32,33]. The Kidney Disease Outcomes Quality Initiative guidelines report that ACR measurement in a first-morning spot urine collection is a reliable method for kidney failure [34].

Given that T2D is the main cause of CKD, it is essential to initiate screening and management programs for diabetic nephropathy, HTN, and CVD globally [35,36]. Therefore, the objective of this study was to examine the relationship between MAU as an indicator of kidney dysfunction and HTN in patients with T2D. It was hypothesized that individuals with T2D who have HTN will have an increased likelihood of having MAU.

## 2. Materials and Methods

### 2.1. Design

This study was a cross-sectional design and the sample consisted of 110 Turkish immigrants with T2D, aged 30 years and older.

### 2.2. Study Population

The participants were recruited from multiple sources in The Hague, The Netherlands. The Hague is vastly urbanized with a large population of Turkish immigrants [21,22]. During a 3-month period, approximately 300 letters written in Dutch and Turkish languages outlining the study were mailed to residents of The Hague with Turkish surnames listed in the local telephone directory. The interested participants could respond to the invitation letter. Due to unknown addresses, 1% of the unopened letters were returned undeliverable. Posters and flyers were also displayed at a family physician’s office, dietitian’s office, health club center, mosque, hairdresser, grocery store, community center, and pharmacies where Turkish immigrants are known to visit. From this, 11 participants were enrolled from the delivered mail, 60 participants were enrolled from the physician’s office, and 20 participants from other sources. A local community representative recruited 19 eligible participants. In this case, 22 potential participants did not qualify for the study because they were either not Turkish (n = 3), or did not have T2D (n = 8), or did not provide blood samples within the 3-month study period (n = 11). Interested participants were initially contacted by phone, at which time the study purpose was explained, and age and gender of the responders were recorded. To ascertain T2D status, each participant was asked for the age at diagnosis and initial treatment modalities. All participants were physician-diagnosed with T2D as having fasting serum glucose level of ≥6.1 mmol/L based on the classification recommended by the Dutch College of General Practitioners [37]. The study was performed according to Dutch legislation regarding Ethics and Human Research and approved by The Central Committee on Research Involving Human Subjects (Centrale Commissie Mensgebonden Onderzoek). Florida International University Institutional Review Board (FIU-IRB) also approved the study protocol (project identification code: 111210-01, date of approval: 12 February 2010). Informed consent was obtained from all participants prior to the commencement of the study.

### 2.3. Study Variables

A research assistant (RA) fluent in both Dutch and Turkish languages interviewed participants in their preferred language. The RA assisted and completed questionnaires on age, smoking status, and prescription medication(s) use including diabetes medications, lipid-lowering drugs (LLD), and hypertension medications. The pilot investigator reviewed the questionnaires, and the participants were contacted to inquire about any missing information. All anthropometric measurements were obtained using standard techniques with the participant wearing light clothing without shoes [38]. Height (to the nearest 0.1 cm) was determined by using a wall-mounted stadiometer, and weight (to the nearest 0.1 kg) was determined by using a digital electronic scale. BMI was calculated as weight (kg) divided by height (m^2^). Blood pressure (BP) was measured according to standard procedure by using an automated office blood pressure equipment [39]. Both systolic blood pressure (SBP) and diastolic blood pressure (DBP) were measured with participants in a sitting position in a quiet room for several minutes while readings were being taken. Measurements were performed twice, and the average reading taken as the individual’s BP. Hypertension was defined as: SBP ≥ 140 mmHg or DBP ≥ 90 mmHg or use of hypertension medications. Smoke status was defined as having serum cotinine levels ≥25 ng/mL according to Medisch Centrum Haaglanden laboratory reference standards. The ACR was calculated to determine the presence of MAU and defined as ACR ≥ 3.5 mg/mmol for females or ACR ≥ 2.5 mg/mmol for males [40].

### 2.4. Laboratory Examination

Venous blood (20 mL) was collected after an 8–12 h overnight fast, by a certified phlebotomist using standard laboratory techniques. Immediately after collection, blood samples were centrifuged, serum/plasma was separated, frozen and stored at −80 °C for analysis. All measurements were performed in a hospital laboratory at Medisch Centrum Haaglanden. Urinary albumin was measured by immunoturbidimetric assay using Tina-quant Albumin, which is hardly influenced by endogenous and exogenous interfering factors [41]. Urinary creatinine was measured by a modified Jaffe method first described in 1886 [42]. Glycated hemoglobin (A1C) was determined by high-pressure liquid chromatography (HPLC) using the Bio-Rad Variant II Turbo Hemoglobin A1C assay. This method is certified by the National Glycohemoglobin Standardization Program (NGSP) and standardized by the International Federation of Clinical Chemistry and Laboratory Medicine (IFCC) [43]. Serum cotinine analysis was performed with an Immulite 1000 system (Siemens Healthcare Diagnostics Products Ltd., Camberley, Surrey, UK), a solid-phase competitive chemiluminescent immunoassay, using the manufacturer’s Nicotine Metabolite Assay kit.

### 2.5. Data Analysis

Chi-square tests were performed to compare percentage differences between participants with positive and negative MAU. Unadjusted and adjusted logistic regression models were conducted to investigate the extent to which HTN is associated with an increased likelihood of having positive MAU. The logistic regression model was adjusted for age, gender, BMI, glycated hemoglobin (A1C), diabetes medications, lipid-lowering drugs (LLD), hypertension medications, and serum cotinine. Statistical analyses were performed using the SPSS, version 19.0 (SPSS Inc., Chicago, IL, USA). Level of statistical significance was set at *p* < 0.05.

## 3. Results

The percentage of participants classified by MAU and related other characteristics are presented in Table 1. MAU was present in 21% (n = 23) of Turkish immigrants with T2D. There was a significantly higher percentage of MAU in males (65.2%) as compared to females (34.8%) (*p* = 0.011). There were a significant higher percentage of participants who tested positive for MAU with A1C ≥ 53 mmol/m (*p* = 0.032), hypertension (*p* = 0.007) and marginally significant for BMI (*p* = 0.052) as compared to those with normal albuminuria.

Unadjusted odds ratio (Table 2) indicated that the odds of having positive MAU were 6.4 times higher in hypertensive T2D individuals compared to those that without HTN (*p* = 0.007; 95% confidence interval [CI]: 1.41, 29.15). Adjusted odds ratio (Table 2) showed that after controlling for covariates, including age (*p* = 0.248), gender (*p* = 0.069), BMI (*p* = 0.073), A1C (*p* = 0.118), LLD (*p* = 0.623), diabetes medications (*p* = 0.715), and serum cotinine (*p* = 0.048), the odds of having positive MAU were 6.6 times higher in hypertensive T2D individuals compared to those that without HTN (*p* = 0.031; 95% confidence interval [CI]: 1.19, 36.39).

## 4. Discussion

The risk for developing MAU is greater among individuals with T2D and existing coronary heart disease [24]. Increased levels of albumin in turn predict the risk for diabetic nephropathy, including loss of kidney function and ESRD [11,44]. Based on such findings, MAU may represent a key diagnostic target for CVD risk among individuals with T2D. Research involving specific ethnic populations, such as the Turkish, suggests that MAU is an important clinical indicator among individuals with diabetes and HTN in predicting CVD risk [20,45,46]. A multinational observational study entitled i-SEARCH, investigated 1926 hypertensive patients from various locations in Turkey to examine the prevalence of MAU and associated risk factors [45]. The high prevalence of MAU and significant cardiovascular risk factors among Turkish diabetic hypertensive patients reported in Kozan’s study and Cöl’s study were similar to that reported in the DEMAND study and PAIT-survey [36,45,46,47]. In these studies, the ACR was determined by dipstick spot urine collection, whereas our results were analyzed in hospital laboratory and measured by immunoturbidimetric assay to determine urinary albumin and Jaffe’s method for estimation of creatinine in serum and urine samples. In contrast with aforementioned studies that were conducted in Turkey, our study investigated first-generation Turkish immigrants living in deprived neighborhoods of The Hague, Netherlands. There is a lack of studies reporting on Turkish immigrants with T2D and HTN living in deprived areas. Dyslipidemia and MAU are known factors to be associated with abdominal obesity. Moreover, cardiovascular and consequently all-cause mortality due to lipid abnormalities could be significantly influenced by kidney dysfunction in T2D patients.

A growing body of research illustrates the importance of MAU as a predictor of CVD risk in the Turkish population. A cross-sectional study of approximately 7708 Turkish individuals with HTN, diabetes, or a combination of the two conditions reported that the prevalence of MAU among these three groups was 17.4%, 20.4%, and 22.1%, respectively [46]. The RiskMan Study Group reported an even greater prevalence of MAU in the Turkish hypertensive population [45]. In this observational, cross-sectional study involving over 1900 hypertensive patients, researchers found the prevalence to be 64.7%. In spite of the known research, which suggests that individuals with HTN have an increased prevalence of MAU, patients with this condition may not be adequately evaluated for CVD risk. While many patients possess one or more known risk factors for CVD, disease risk management was inadequate. Furthermore, a majority of the patients failed to comply with antihypertensive medication regimens. Similarly, concerning findings were observed among those subjects who also had diabetes.

As compared to the native Dutch population, the Turkish immigrants in the Netherlands show (i) higher prevalence of T2D, (ii) higher prevalence of CVD, and (iii) lower prevalence of HTN [13,18,48]. The lower prevalence of HTN among the Turkish could be due to awareness and increased treatment levels with hypertension medications. Nevertheless, our study revealed that hypertensive Turkish individuals with T2D were more likely to have elevated ACR compared to those that were normotensive. Additionally, positive MAU was present in 21% of our study population increasing their risk for CVD. In the Systolic Hypertension in the Elderly Program, SBP was a strong predictor of decline in renal function [49]. In the Framingham study, pulse pressure predicted CVD in individuals aged more than 60 years, whereas DBP was the strongest predictor in those aged more than 50 years [50]. Research indicates a higher prevalence of MAU in individuals with HTN and observed a significant relationship between ambulatory BP and ACR [51].

The results of this study suggest that individuals with HTN have a significantly higher percentage of having positive MAU compared to normotensives. A prospective, long-term follow-up study, conducted on 574 patients with T2D indicated that risk factors including BP, BMI, A1C levels, male gender, and plasma cholesterol categorize individuals for poor renal and adverse CVD events [52]. Several studies showed that in hypertensive individuals with T2D, tight BP control could reduce the risk for diabetic nephropathy [11,53].

Various studies have reported remission and/or regression of MAU in individuals with T2D [54,55]. Araki et al. reported remission/regression of MAU in approximately 50% of individuals with T2D in a 6-year prospective study [54]. Antihypertensive therapy and improved glycemic control were independent predictors for remission to normoalbuminuria in individuals with T2D [55]. A prospective study conducted by Mogensen et al. revealed that ACR decreased by 50% with a combination treatment of antihypertensives [56]. Furthermore, the authors suggested the beneficial therapeutic control of BP for the prevention of diabetic nephropathy and CVD.

Various mechanisms have been suggested for MAU measurements and the best method to identify MAU has yet to be determined. It remains unclear whether microalbumin measurement alone is a sufficient screening method or a calculation of an ACR is necessary to detect microalbumineric hypertensive patients. Published data have shown that the calculation of an ACR exhibited a higher sensitivity and specificity in the detection of microalbuminuria in various groups [9]. However, other studies concluded that the ACR did not provide any advantage compared with microalbumin measurement alone [29]. Additional disagreement exists over the most appropriate cut-off levels of albumin to indicate MAU, as some studies suggest that lower levels of albumin may be predictive of CVD. Several studies showed a good correlation between MAU measurement alone and ACR in spot urine collection and 24-hour urine collection [45,46,47,51,55]. Gender specific cut-off values are recommended for ACR and it is recommended that a positive result needs confirmation by 24-hour urine collection [29]. However, research findings demonstrated a higher sensitivity of ACR than MAU measurement alone [57].

Our study has several strengths. Participants included were all of Turkish origin, a single origin, in which research concerning MAU, HTN, T2D and CVD are limited. We used a standardized protocol, including uniform anthropometric and biochemical measurements in a clinical setting. We adjusted the analysis for all major confounders of MAU such as age, gender, BMI, A1C, LLD, diabetes medications, hypertension medications, and smoking.

There were a number of limitations for this study. First, because of the study’s cross-sectional nature, our results do not establish causality and cannot be generalized for other populations. Our sample was not randomly selected and may not represent all of the Turkish immigrant population of the Netherlands. Second, given the small sample size (n = 110), this study’s findings need to be tested with a larger sample size.

Maintaining adequate BP is an important therapeutic goal among individuals with T2D. The early detection of MAU among members of the Turkish population with HTN is fundamental in preventing and treating diabetes complications and improving individuals’ renal and CVD outcomes. A greater awareness of the importance and utility of MAU detection in evaluating CVD risk is paramount.

## 5. Conclusions

The present study shows that MAU is significantly associated with HTN in Turkish immigrants with T2D living in deprived neighborhoods of The Hague, Netherlands. Further studies are needed to confirm our results and to examine other risk factors such as dietary intake that affect BP levels in individuals with T2D. Research findings can contribute to the management of renal dysfunction and risk factors for CVD in Turkish patients with T2D. Adding MAU testing to standard diabetes care may be used for determining the potential risk for CVD and may direct treatment in the primary prevention of CVD. Further research should be conducted following cohorts to determine the effects of MAU and HTN for person with T2D on CVD morbidity and mortality.

## Figures and Tables

**Table 1:** Percentages of participants, classified by microalbuminuria and related other characteristics for Turkish immigrants with type 2 diabetes in The Hague, Netherlands, 2011.

	Microalbuminuria		
Variables	Positive * (n = 23)	Negative (n = 87)	*p* Value
Age (years) (%)			0.194
<55	56.6	41.4	
≥55	43.5	58.6	
Gender (%)			**0.011**
Male	65.2	35.6	
Female	34.8	64.4	
Body mass index (kg/m^2^) (%)			0.052
<30	17.4	39.1	
≥30	82.6	60.9	
Glycated hemoglobin (mmol/m) (%)			**0.032**
<53	43.5	67.8	
≥53	56.5	32.2	
Diabetes medications (%)			0.336
No	34.8	46.0	
Yes	65.2	54.0	
Lipid lowering drugs (%)			0.545
No	43.5	50.6	
Yes	56.5	49.4	
Serum cotinine (ng/mL) (%)			0.189
<25	60.9	74.7	
≥25	39.1	25.3	
Hypertension (%)			**0.007**
Normotensive	8.7	37.9	
Hypertensive	91.3	62.1	
Total cholesterol (mg/dL) (%)			-
<8	0	0	
≥8	0	0	
High-density lipoprotein (mg/dL) (%)			0.942
≥1.7	8.7	9.2	
<1.7	91.3	90.8	
Low-density lipoprotein (mg/dL) (%)			0.942
<4.4	8.7	9.2	
≥4.4	91.3	90.8	
Triglycerides (mg/dL) (%)			0.252
<2.1	34.8	23.0	
≥2.1	65.2	77.0	
Urine Creatinine (mmol/L) (%)			0.081
<18 for males and <16 for females	4.3	19.5	
≥18 for males and ≥16 for females	95.7	80.5	

Notes: * Positive microalbuminuria is defined as albumin-to-creatinine ratio ≥ 3.5 mg/mmol for females and/or albumin-to-creatinine ratio ≥ 2.5 mg/mmol for males. Hypertensive was defined as: systolic blood pressure ≥ 140 mmHg or diastolic blood pressure ≥ 90 mmHg or use of hypertension medications. Bold: *p*-value is considered significant at <0.05.

**Table 2:** Unadjusted and adjusted * odds ratios of having microalbuminuria by hypertension status.

	Unadjusted			Adjusted *		
Parameter	OR	95% CI	*p* Value	OR	95% CI	*p* Value
Normotensive ^a^	1.00			1.00		
Hypertensive	6.42	1.41, 29.15	**0.007**	6.58	1.19, 36.39	**0.031**

Abbreviations: CI, confidence interval; OR, odds ratio. ^a^ Reference category for *p*-value. Notes: * Covariates included in the logistic regression analysis were age (*p* = 0.248), gender (*p* = 0.069), body mass index (*p* = 0.073), glycated hemoglobin (*p* = 0.118), diabetes medications (*p* = 0.715), lipid-lowering drugs (*p* = 0.623), and serum cotinine (*p* = 0.048). Microalbumiuria is defined as albumin-to-creatinine ratio ≥ 3.5 mg/mmol for females and/or albumin-to-creatinine ratio ≥ 2.5 mg/mmol for males. Hypertensive was defined as: systolic blood pressure ≥ 140 mmHg or diastolic blood pressure ≥ 90 mmHg or use of hypertension medications. Bold: *p*-value is considered significant at <0.05.

## Data Availability

The raw data supporting the conclusions of this article will be made available by the authors, without undue reservation.
